# Integrating genomic prediction into crop DUS testing: new approaches in support of reference collection management and distinctness assessment

**DOI:** 10.1007/s00122-026-05198-6

**Published:** 2026-03-12

**Authors:** Adrian M. I. Roberts, Konrad Neugebauer, Esther Oluwada Ewaoluwagbemiga, Dan Milbourne, Stephen Byrne, James Cockram, Camila M. Zanella, Margaret Wallace, Marlene Niedermayer, Lorella Andreani, Márton Pécs, Wim W. P. van der Kooij, Karl Schmid

**Affiliations:** 1 Biomathematics and Statistics Scotland (BioSS), Edinburgh, UK; 2 Humboldt-Universität zu Berlin, Berlin, Germany; 3 Biomathematics and Statistics Scotland (BioSS), Dundee, UK; 4 University of Edinburgh, Edinburgh, UK; 5 Teagasc, Carlow, Republic of Ireland; 6 Niab, Cambridge, UK; 7 Austrian Agency for Health and Food Safety (AGES), Vienna, Austria; 8 Council for Agricultural Research and Economics - Research Centre for Plant protection and Certification (CREA), Tavazzano, Italy; 9 National Food Chain Safety Office (NÉBIH), Budapest, Hungary; 10 Naktuinbouw, Roelofarendsveen, The Netherlands; 11 University of Hohenheim, Stuttgart, Germany

## Abstract

**Key message:**

**A new approach is proposed for the use of the genetic markers to manage DUS trials, targeted at individual phenotypic characteristics using genomic prediction, as well for supporting Distinctness decisions.**

**Abstract:**

High-performing crop varieties underpin food security. Due to the cost of developing varieties, systems have been established to provide breeders with legal protection for their varieties. In many countries, such protection is afforded by the International Union for the Protection of New Varieties of Plants (UPOV) system. New varieties must be phenotypically Distinct from existing varieties using a set of crop-specific characteristics, as well as Uniform and Stable (DUS). For many crops, DUS assessment is costly as candidates must be compared to many existing varieties in field trials, based on numerous DUS characteristics. The use of genetic markers has long been considered as a potential tool for managing costs of such trials, for example, by identifying existing varieties that need not be compared to candidate varieties. Under UPOV guidance, the use of genetic markers must be reflective of phenotypic differences in DUS characteristics. Within this framework, we propose a new approach for using markers based on the application of genomic prediction, which is used to predict variety differences in individual characteristics. The approach is evaluated with perennial ryegrass and wheat, yielding promising results. Additionally, we propose a novel approach in which genomic prediction is used to refine Distinctness decisions after DUS trials have been run by integrating genetic and trial information. Using perennial ryegrass as an example, we demonstrate that this approach, which respects the primacy of phenotype in DUS testing, could be used to support distinctness decisions, especially for cross-pollinated agricultural crops where Distinctness may be harder to achieve.

**Supplementary Information:**

The online version contains supplementary material available at 10.1007/s00122-026-05198-6.

## Introduction

Development of new high-performing varieties is critical for food security, but is costly. Breeding is incentivised if this investment is safeguarded through legal protection. The International Convention for the Protection of New Varieties of Plants established principles of plant variety protection and institutes a treaty body called the International Union for the Protection of New Varieties of Plants (UPOV). In 2025, UPOV comprised 80 member states and intergovernmental organisations, which had acceded to the convention. According to the convention, new plant varieties must be found to be Distinct, Uniform, and Stable (DUS) to qualify for protection (www.upov.int), based on phenotypic assessment of an agreed set of crop-specific traits, termed ‘DUS characteristics’.

Within a given country, DUS assessment is carried out by or on behalf of the Authority who has the right to grant breeders’ rights (according to the 1991 Act of UPOV Convention). New ‘candidate’ varieties submitted by breeders to an Authority must be demonstrated to be Distinct from any other variety whose existence is a matter of common knowledge (UPOV 2002). Distinctness is generally established through a growing test, often conducted in field or glasshouse trials, and assessments of specific DUS characteristics. The approach for assessing Distinctness depends on the type of characteristic, as well as the species of crop and method of propagation (UPOV 2002). Where expression of characteristics for a variety is sufficiently consistent, records are made from visual assessments. In other cases, statistical means such as analysis of variance may be used for measured characteristics.

For established species the number of varieties in ‘common knowledge’ as defined by the UPOV Members can be very large. Testing facilities create a variety collection, also known as ‘Reference Collection’, which can be much smaller and is comprised of only those varieties of common knowledge relevant to the Authority (UPOV 2008). However, in the case of established crops, Reference Collections can still be extensive, consisting of many hundreds of varieties. Candidate varieties must demonstrate Distinctness from each variety of common knowledge. Distinctness is determined based on a specific set of physically expressed DUS characteristics, commonly including morphology, colour, phenology, and disease resistance. UPOV provides Test Guidelines for many crops that define the set of characteristics (https://www.upov.int/test_guidelines/en/list.jsp). The guidelines are open to interpretation and there are differing techniques to manage the size of the growing trials. For some cross-pollinated species, in the absence of prior information regarding such Distinctness, candidates must be compared against all varieties in the Reference Collection even in the first year of field trials. In other cases, where expression of characteristics is sufficiently consistent, the testing process may be different. For instance, in the first year of trial a preliminary DUS description of candidates can be produced and this used to select similar varieties that need to be compared in a growing trial in the subsequent year(s). Information prior to the first year of DUS testing may be derived from responses to a Technical Questionnaire provided by the applicant at the point of submission of the candidate variety for DUS testing. However, this information is only reliable for certain DUS characteristics that are consistently expressed and readily assessed, for example, the DUS character ‘Ear: number of rows’ in the cereal crop barley (UPOV 2020b). Additionally, many agricultural species have insufficient characteristics of such nature to make reliable a priori decisions, necessitating the assessment of Distinctness in extensive and costly DUS trials.

Genetic markers provide an additional route to Reference Collection management, that is the identification of which varieties candidates need to be compared with in the DUS trials. The aim is a more efficient DUS trial system, either through reduction in the number of known varieties that need to be sown, or by earlier identification of the most similar varieties to allow more effective trial design. The use of genetic markers in DUS assessment has been extensively discussed in UPOV technical working parties over a number of years, as well as external forums (e.g., Camlin 2003; Jones et al. 2013a; Gilliland et al. 2020; Jamali et al. 2020; Yang et al. 2021; Yu and Chung 2021), including proposals that genomic information might be directly employed for Distinctness decisions. However, to date, UPOV has agreed that molecular markers can only be utilised when linked to the phenotype, either as a compositive distance or directly to phenotypic characteristics used within the existing DUS system.

The current guidance (UPOV 2020a) describes two application models for the utilisation of molecular markers in the examination of DUS:

- Characteristic-Specific Molecular Markers
- Combining Phenotypic and Molecular Distances in the Management of Variety Collections

UPOV application model a) is a useful approach for crops where there are DUS characteristics for which genetic markers can be found that predict exactly the expression of the trait (either partially or completely), with the theoretical example of herbicide resistance being given by UPOV in general guidance (2020a), and with practical examples found in individual crop Test Guidelines. While molecular markers with high marker-trait associations have been found for DUS characteristics in various crops (e.g., for wheat: Yang et al. 2021; and barley: Cockram et al. 2010, 2012; Saccomanno et al. 2020), the applicability of this model is limited, as many crops include few or no such characteristics in their DUS Test Guidelines.

To enhance the efficacy of trial management, it is beneficial to use a wider range of DUS characteristics, such as under UPOV application model b). A possible criticism of the current methods described under this model is that they are not targeted to individual DUS characteristics: instead of comparing genetic distance to differences in individual characteristics, an overall phenotypic distance is employed, combining scores over characteristics. However, Distinctness is assessed for characteristics individually, rather being based on multivariate comparisons. Furthermore, the method also requires a reasonably strong correlation between genetic distances and phenotypic distances (across all characteristics). This correlation varies among crops (e.g., Kwon et al. 2005; Gunjaca et al. 2008; Wang et al. 2023; Liu et al. 2022; Hong et al. 2021; Julier et al. 2018). Whilst low correlations can be due to insufficient genetic markers (Jones et al. 2013b), in some crops the relationship between phenotypic and genotypic distances may be inadequate for use in Reference Collection management, with Jones and Mackay (2015) suggesting a minimum correlation of 0.6 is necessary to avoid errors in Distinctness decisions.

Genomic prediction is a set of tools aimed at utilising arrays of genome-wide markers to predict trait values given the genotype. Genomic Prediction has been a rich area for research and numerous methods have been developed, including Genomic Best Linear Unbiased Prediction (gBLUP), the Bayesian alphabet methods, as well as machine learning approaches (Sorensen 2023; Crossa et al. 2017; Alemu et al. 2024; Lourenço et al. 2024; Chafai et al. 2023). The proposed approach for Reference Collection management is a framework in which any suitable genomic prediction method could be applied, with the choice depending on performance. The relative performance of genomic methods is contingent on the complexity of the genetic architecture by which the trait is controlled (Meher et al. 2022). Certain methods, such as gBLUP, work well when there are many underlying quantitative trait loci (QTLs) with small phenotypic effects, whereas others, such as Bayesian Lasso, work better when there are a smaller number of QTLs with larger phenotypic effects. The methods also vary in computational cost, with Bayesian methods in particular incurring a substantial computational burden.

This study proposes a new approach based on genomic prediction to enhance Reference Collection management, as well as Distinctness assessment for specific types of crops.

### Reference collection management with genomic prediction

Our approach for Reference Collection management integrates the advantages of a targeted linkage between genetic markers and DUS characteristics of UPOV application model a), with the comprehensive coverage of all DUS characteristics of UPOV application model b). Genomic prediction underpins this framework, following work by Jones and Mackay (2015). It is hypothesised that this approach should surpass the performance of UPOV application model b) since it uses the genetic information in a more driven manner. The theoretical framework in which our approach was designed is particularly suitable for DUS characteristics measured on a more quantitative scale as it enables the identification of decision thresholds predicted to distinguish varieties correctly, even if the genetic marker-based prediction of the mean is with error. The proposed approach is evaluated using historical DUS data sets for two agricultural species, wheat (*Triticum aestivum* L., a predominantly inbreeding broadacre crop grown throughout the world and a cornerstone of global food security), and perennial ryegrass (*Lolium perenne* L., an outbreeding species cultivated for animal grazing and fodder, and used as a hard-wearing turf for gardens and golf courses).

Genomic prediction is used prior to undertaking the DUS trial, to predict the difference in a specific DUS characteristic between a ‘candidate’ variety, which lacks DUS field data, and a ‘known’ variety, which has historical DUS field data. This information can subsequently be employed to manage the DUS trial in crops such as perennial ryegrass or oilseed rape, by identifying the subset of known varieties that are highly likely to be Distinct from the candidate, thus eliminating the need for field comparison. Alternatively, for many other crops such as barley, maize, and wheat, it can be utilised to identify the most phenotypically similar varieties for close comparison in the first year of DUS trials. Under the system proposed here, whilst the known varieties would have both historical DUS phenotypic data and genetic marker data, the candidate would only have marker data at this stage of the assessment. The genomic prediction model is built based on multiple years’ historical DUS data encompassing the Reference Collection, and ideally accounts for variation between trials as well as the genetic component. With many forms of genomic prediction, it is not only possible to estimate the difference in mean between the candidate and the known variety, but also to calculate a standard error for this difference. Making reasonable assumptions about distributions, the statistical significance of this difference can be evaluated, for example by comparison with Student’s t-distribution, giving a basis for determining whether the two varieties are likely to be considered Distinct. The same probability threshold can be employed as for the UPOV Combined-Over-Years Distinctness COYD criterion (COYD), being typically 0.01. COYD applies analysis of variance to individual characteristics, using Student’s t-test to assess the significance of differences between pairs of varieties (Patterson and Weatherup 1984; UPOV 2022). Such assessments of gBLUP ‘Distinctness’ for a particular pair of varieties can be collated over all DUS characteristics, adhering to the doctrine that Distinctness is only required in one DUS characteristic.

### Distinctness assessment with genomic prediction

In addition to its use for Reference Collection management, we propose that genomic prediction can be applied to enhance Distinctness assessment for candidates with field data, with a method designed for cross-pollinated crops with measured quantitative characteristics. Other than for Reference Collection management following UPOV guidance (UPOV 2020a), it is not currently possible to directly assess Distinctness using genetic markers alone, as proposed by e.g., Yang et al. (2021), without reference to the UPOV DUS characteristics (UPOV FAQs). However, UPOV application model a) indicates a use for markers in determining Distinctness for characteristics that can be precisely predicted. Our proposal uses genetic marker information in a different way, to enhance the precision of mean estimates in individual DUS characteristics that underpin Distinctness decisions.

The new approach for assessment of Distinctness works by utilising information from the genetic markers to augment COYD. Unlike the application of genomic prediction to Reference Collection management, here both candidate and known varieties have DUS trial data. The added information should improve the estimation of means of the phenotypic characteristics, thereby refining Distinctness decisions using a COYD-like approach. The approach is suitable for application in cross-pollinated crops with measured quantitative characteristics where COYD is already employed, such as perennial ryegrass or alfalfa (*Medicago sativa* L.). We designate this method COYD-GP and it is demonstrated utilising a historical DUS data set for perennial ryegrass. COYD-GP uses an equivalent linear mixed model, but with a random effect for variety with covariances specified through a genetic relationship matrix, computed from the genetic markers.

## Materials and methods

### Example data-sets

DUS phenotypic and genetic data sets were compiled for perennial ryegrass and wheat. Historical phenotypic data derived from DUS trials and samples of plant material were supplied by European Union (EU) and United Kingdom (UK) DUS testing organisations, following European protocols (CPVO Technical Protocols 2025) based on UPOV Technical Guidelines. Under a data sharing agreement, access was granted exclusively for registered varieties with the permission of their owners. Consequently, the number of varieties available for this study was significantly limited, to approximately 50% of registered varieties in the case of perennial ryegrass. Variety names were encoded to ensure confidentiality. For the analyses reported herein, only varieties possessing both phenotypic and genotypic data were considered.

#### Perennial ryegrass

The study encompassed 119 diploid and 149 tetraploid varieties of forage perennial ryegrass, thus totalling 268 varieties. Trait data was obtained from DUS trials conducted at Naktuinbouw, Netherlands, over a 13-year period from 2007 to 2019. Twenty-one DUS characteristics were measured in one DUS trial per year (UPOV 2006). The analysis used trial means for each variety; within-trial data was not available. These measurements were expressed in standard units (e.g., cm for height) rather than UPOV notes (scores from 1 to 9). All DUS characteristics examined were quantitative in nature (Supplementary Information).

Seed from each variety was germinated in a glasshouse in small pots and a lawn was allowed to establish. The lawn was then cut to approximately 3 cm and the cuttings discarded, and the lower 3 cm was harvested into envelopes and freeze dried. Freeze dried material from each cultivar was transferred into 50 ml falcon tubes and milled to a fine powder using steel balls on a RETSCH Mixer mill. Genomic DNA was then extracted using a CTAB protocol, and varieties were genotyped with a genotyping-by-sequencing (GBS) approach, utilising restriction enzymes to reduce genome complexity (Elshire et al. 2011). The restriction enzymes used for genome complexity reduction were *Pst*I and *Msp*I and libraries were prepared and sequenced using Illumina paired-end 150-bp reads by LGC Genomics (Berlin, Germany). The reads were aligned to the reference genome (Nagy et al. 2022) using Burrows-Wheeler Aligner (BWA) (Li 2013) and variant sites identified using bcftools mpileup and call (Danecek et al. 2021; Li 2011). From an initial set of 1.5 million sites, a high-quality set of 187k single nucleotide polymorphisms (SNPs) was identified after applying the following filtering criteria: minimum read depth of 30 for a sample; ≤ 9.9% of samples missing at a site; average variant allele frequency between 0.05 and 0.95. The genome resequencing reads for all perennial ryegrass cultivars utilised in this study have been deposited into the NCBI sequence read archive (SRA) under the BioProject ID: PRJNA1255563.

Allele frequencies were used in computations (de Bem Oliveira et al. 2019), based on the number of reads for the non-reference allele over the total number of reads. A genetic relationship matrix (GRM) was computed in R (R Core Team 2022), utilising the Gmatrix function from the R package AGHmatrix (Amadeu et al. 2023) based on additive effects, employing the approach of Van Raden (2008), adapted for allelic frequencies (Cericola et al. 2018). To accommodate mixed ploidies, the sampling variance was employed as a correction factor rather than using a parametric correction.

A Genome-Wide Association Study (GWAS) (Uffelmann et al. 2021), was conducted to identify SNPs linked to quantitative trait loci (QTL) based on the perennial ryegrass data set. The overall genetic sub-structure of the set of varieties was accounted for by the inclusion of the GRM in the model. A linear mixed model was fitted with fixed effects for ploidy and the specific SNP, and random effects for variety (using the GRM to specify covariance between the varieties), year and the interaction between year and ploidy. The GWAS function of the R package sommer was used, which has an efficient algorithm for large numbers of genetic markers (Covarrubias-Pazaran 2016). Two significance thresholds were used: the Bonferroni correction, with the limit set to achieve an overall *p*-value of 0.05; and the False Discovery Rate (FDR) method (Benjamini and Hochberg 1995), using the R function p.adjust (in R statistics library), and thresholding the adjusted p-value at 0.05. Given the paucity of significant genetic markers identified, SNPs just below the Bonferroni threshold were also considered. For characteristics where potential QTLs were found on multiple chromosomes, a forward selection process was adopted.

#### Wheat

Historical DUS data for 27 CPVO DUS characteristics for 423 wheat varieties were supplied by testing organisations for the UK, Germany, Hungary, Austria, and Italy, as previously described by Zanella et al. (2026). Briefly, for each DUS character, DUS phenotypic data was recorded as UPOV notes (1 to 9 scale), with one data point per variety in each county summarising the DUS phenotype as assessed over at least two years of DUS testing. Similarly, the wheat genotypic dataset was also sourced from Zanella et al. (2026). Briefly, for each accession a single seed was grown, genomic DNA extracted, and genotyping undertaken using the ‘*Triticum aestivum* Next Generation’ (TaNG) 43k Axiom array (Burridge et al. 2024). The resulting genotypic dataset was processed to remove markers with missing data ≥ 10% and markers with a minor allele frequency ≤ 3%, and the remaining missing values in the genotypic data were imputed using the R package missForest (Stekhoven and Buehlmann 2012). Here, we focussed on 19 quantitative DUS characteristics (UPOV 2017). Comparisons were limited to varieties trialled in the same country, owing to few varieties being tested by more than one country.

GWAS was performed in R version 4.4.1 using the package GWASpoly (Rosyara et al. 2016) which implements a linear mixed model. Population genetic stratification was accounted for using (i) population structure as a fixed effect, via the use of principle components (PCs), and (ii) kinship as a random effect, determined using a subset of 4,252 SNPs skimmed from the final genotypic dataset using a correlation threshold of 0.60. Bonferroni correction and FDR with a *q*-value cut-off of *q* = 0.05 was used to identify marker-trait associations. A GRM was computed with the GWASpoly package. GWASpoly uses a random polygenic effect to control for population structure, which is called the K model in the GWAS literature (Yu et al. 2006).

### Reference collection management

To enable a comparison of the new approach with the current UPOV application model b), the datasets described above were used to calculate genetic and phenotypic distances. Rogers’ distance (Rogers 1972) was used for the genetic distance, and phenotypic distance was based on quantitative characteristics only using a scaled Euclidean distance.

To illustrate the new approach, the most straightforward genomic prediction method, genomic best linear unbiased prediction (gBLUP) was used (Isik et al. 2017). gBLUP relies solely on the pairwise genetic relationships between varieties, calculated from the markers, and represented in the Genetic Relationship Matrix (GRM). The gBLUP model is a linear mixed model incorporating a random effect for variety (specifying the covariances through the GRM). Additional effects may be included to reflect data structure, typically year (or trial) as a random effect. For perennial ryegrass, a fixed effect for ploidy was included, as well as a random effect for the year-by-ploidy interaction. For wheat, where DUS data were available for varieties assessed in several countries, the model incorporated random effects for country and the interaction between variety and country. In this case, comparisons between varieties were made only within countries, given that both trial conditions and the genetic base were likely to change substantially between countries (and there were few varieties in common between the countries). For DUS characteristics, where genetic markers linked to QTL were identified using GWAS, the standard gBLUP model was augmented to incorporate fixed effects for these markers, referred here as gBLUP + QTL (c.f. Bernardo 2014), maintaining the same GRM. The demonstration of this methodology has been limited here to non-binary quantitative characteristics. Binary traits are more appropriately dealt with via UPOV application model a). Linear mixed model analyses, including gBLUP and gBLUP + QTL were carried out using the ASREML-R package (Butler et al. 2017) within R.

For each of the example data sets, we evaluated the potential practical utility of the approach. Performance was assessed utilising a leave-one-out cross-validation scheme designed to simulate the intended use in Reference Collection management, where each variety in turn was treated as a ‘candidate’. A gBLUP model was fitted to the remaining varieties, which were treated as ‘known’ varieties, and disregarding the phenotypic data for the ‘candidate’. Based on this model, predictions were generated for the differences in over-years characteristic means between the candidate and each of the ‘known’ varieties. An evaluation was made as to whether each pair was predicted to be considered Distinct based on Student’s t-test with probability level of 0.01. This test was approximated to a normal test as the ASREML-R package does not estimate degrees of freedom for contrasts of random effects. The degrees of freedom were certain to be above 100 for both data sets, so the approximation is good.

For ryegrass, these predictions were compared to differences from a long-term COYD analysis (UPOV 2022) of the complete data set. The long-term COYD analysis was based on a mixed model similar to the gBLUP model, except the variety effect was a simple random effect without genetic information. These COYD differences were compared to a least significant difference based on three years of data with a probability level of 0.01 for ryegrass. For wheat, the predicted differences were compared to the actual differences (with only one value supplied for each variety), with a difference of at least two notes indicating Distinctness. The predicted differences using either gBLUP or gBLUP + QTL were collated across all characteristics to obtain an overall assessment of the approach’s effectiveness.

The potential trial size reduction for perennial ryegrass given the proportions of varieties discriminated by genomic prediction in the cross-validation study was assessed by simulation.

If it is assumed that the whole Reference Collection must be planted unless all the candidates are considered to be Distinct from some of the known varieties, then we can estimate the potential trial size reduction through simulation. A random sample of the varieties is treated as a set of candidates, and subsequently the proportion of ‘known’ varieties that are Distinct from all of these ‘candidates’ is calculated. The mean of this proportion over 1000 simulated samples provides an estimate of the expected reduction in trial size afforded by genomic prediction.

### Using genomic prediction to enhance distinctness decisions (COYD-GP)

COYD-GP was demonstrated using the perennial ryegrass data set. For each characteristic, the number of differences between each pair of varieties was determined based on a long-term version of COYD (UPOV 2022), and compared to results using a long-term version of COYD-GP. A 1% probability value is utilised to threshold Distinctness decisions. The long-term versions of COYD and COYD-GP applied were similar to those which would be employed in practice, except that the whole historical data set was utilised, rather than a specific set of two or three years that might typically be used for Distinctness assessment. An additional difference was the use of a linear mixed model used to represent COYD, as opposed to analysis of variance. Variety, year, and year-by-ploidy are treated as random effects, with ploidy being a fixed effect. Comparisons were conducted between varieties in the same ploidy group.

## Results

### GWAS

We present GWAS results only briefly here as the focus of this study was on demonstrating the proposed framework for Reference Collection management. For perennial ryegrass, SNPs were found to be potentially linked to QTLs using GWAS for five of the 21 DUS characteristics analysed: ‘Plant: width (after vernalization)’, ‘Plant: vegetative growth habit (after vernalization)’, ‘Plant: height (after vernalization)’, ‘Plant: time of inflorescence emergence’, and ‘Flag leaf: length/ width ratio’ (Supplementary Information Table S1). For wheat, genetic markers previously identified by Zanella et al. (2026) as being linked closely to potential QTLs for six of the 19 quantitative characteristics: ‘Seed: colouration with phenol’, ‘Coleoptile: anthocyanin colouration’, ‘Ear: density’, ‘Ear: length of scurs or awns’, ‘Lower glume: length of beak’, and ‘Lower glume: area of hairiness on internal surface’ (Supplementary Information Table S2).

### Performance of the current form of UPOV application model b) for reference collection management

**Fig. 1:**
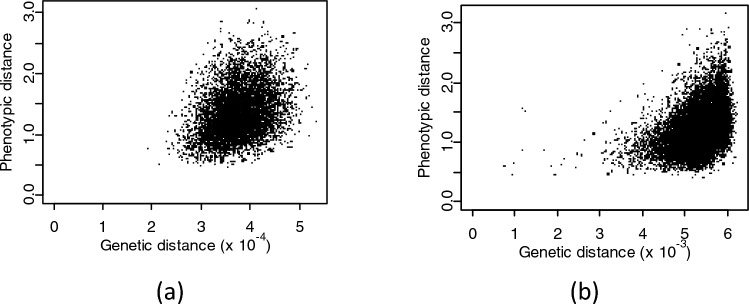
Comparison of phenotypic and genetic distances for pairs of varieties for **a** diploid perennial ryegrass (*Lolium perenne* L.) and **b** hexaploid wheat (*Triticum aestivum* L.). Phenotypic distance is calculated over quantitative DUS characteristics using a scaled Euclidean distance. Genetic distances use Roger’s distance

Figure 1 illustrates the relationships between phenotypic distance and genetic distance for the diploid perennial ryegrass and hexaploid wheat data sets, based on quantitative DUS characteristics only. The correlations were 0.30 for diploid ryegrass, 0.46 for tetraploid ryegrass, and 0.40 for wheat. Such poor correlations highlight the challenges of specifying a safe threshold for genetic distance that would allow identification of pairs of varieties which would be deemed Distinct based on DUS characteristics.

### Performance of genomic prediction for reference collection management

We present performance evaluations of genomic prediction for Reference Collection management based on perennial ryegrass and wheat historical data sets. Firstly, we assessed the ability of genomic prediction to forecast differences between a candidate and known varieties. Then, for perennial ryegrass, we examined how effective these predictions might be in terms of reducing the number of known varieties that need to be included in the DUS trial. For wheat, the objective is to identify close comparisons in advance of trialling. Whilst this may reduce trial size, this calculation based on simulation is less relevant due to the complexity of selection process.

#### Perennial ryegrass

Residual plots for each characteristic based on the gBLUP model without QTLs are given in the Supplementary Information (histogram of residual, normal Q-Q plot, residuals vs fitted value).

**Fig. 2:**
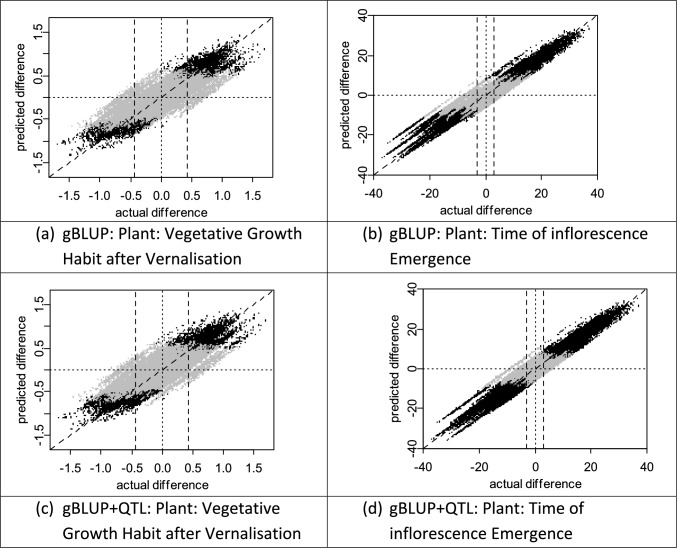
Perennial ryegrass: simulated use of genomic prediction to identify Distinct pairs of varieties for two selected DUS characteristics. The vertical axis represents the differences in predicted mean expression between simulated ‘candidates’, whose phenotypic data is ignored, and all other varieties in the historical data set, representing known varieties. The horizontal axis presents the differences between the same pairs of varieties, but based on the phenotypic data only. Points on the plots represent differences in means between a pair of varieties. The black points are those where genomic prediction finds the candidate and known variety to be Distinct (with probability 0.01) and grey points represent those that were not found Distinct. The vertical dashed lines represent the differences that would be sufficient to be Distinct using the long-term version of COYD (with probability 0.01). The left-hand panels are for DUS characteristic ‘Plant: Vegetative Growth Habit after Vernalisation’, and the right-hand panels are for ‘Plant: Time of Inflorescence Emergence’. The upper panels used gBLUP, whereas the lower panels used gBLUP + QTL

The results of the cross-validation scheme for simulating a real-life application of genomic prediction for two perennial ryegrass DUS characteristics are shown in Fig. 2. Predictions were better for ‘Plant: time of inflorescence emergence’ (R^2^ value of 92.62%) compared to ‘Plant: vegetative growth habit’ (after vernalization)’ (R^2^ value of 71.82%). This characteristic also discriminated more pairs of varieties using field data with long-term COYD (Table 1).

**Table 1:** Perennial ryegrass: proportion of pairs of simulated ‘candidate’ and ‘known’ varieties distinguished by genomic prediction via cross-validated gBLUP and gBLUP + QTL models, compared with long-term COYD results

Ryegrass DUS Characteristic	Diploid			Tetraploid		
	COYD (%)	gBLUP (%)	gBLUP + QTL (%)	COYD (%)	gBLUP (%)	gBLUP + QTL (%)
Plant: vegetative growth habit (without vernalization)	17.6	2.4		6.1	0.4	
Leaf: intensity of green colour (without vernalization)	0.7	0.3		0.1	0.1	
Plant: width (after vernalization)	16.3	0.2	1.0	14.3	0.7	2.9
Plant: vegetative growth habit (after vernalization)	30.0	7.2	9.4	19.0	6.5	9.0
Plant: height (after vernalization)	40.7	11.0	13.2	23.7	6.5	8.8
Leaf: intensity of green colour (after vernalization)	7.2	1.2		6.8	4.1	
Plant: time of inflorescence emergence	72.1	24.4	38.6	69.1	27.0	39.0
Plant: natural height at inflorescence emergence	28.3	5.3		20.7	4.8	
Plant: growth habit at inflorescence emergence	9.7	2.9		17.0	12.5	
Flag leaf: length	9.7	0.7		2.9	1.0	
Flag leaf: width	26.0	6.5		21.8	9.6	
Flag leaf: length/ width ratio	10.8	3.7	5.1	3.0	1.1	2.4
Plant: length of longest stem, inflorescence included (when fully expanded)	29.0	7.7		24.3	9.2	
Plant: length of upper internode	4.0	1.1		1.3	0.6	
Inflorescence: length	14.1	0.7		19.9	4.8	
Inflorescence: number of spikelets	23.2	2.6		13.0	1.4	
Inflorescence: density	16.7	2.1		22.1	5.6	
Inflorescence: length of outer glume on basal spikelet	15.5	2.0		10.5	0.1	
Inflorescence: length of basal spikelet excluding awn	10.9	2.2		9.9	4.1	
Inflorescence: spikelet protuberance	18.4	1.6		13.0	3.8	
Inflorescence: glume span	27.9	4.4		12.7	2.0	

In the cross-validation scheme, ‘candidates’ only have genetic data whereas ‘known’ varieties have both phenotypic and genetic data. Results for diploid and tetraploid perennial ryegrass are shown separately

The proportion of pairs of varieties (simulated candidates and known varieties) for each DUS characteristic distinguished by genomic prediction and long-term COYD are shown in Table 1. For all DUS characteristics, results are shown for the gBLUP method, and for those five characteristics with SNPs potentially linked to QTL, results are also given for the gBLUP + QTL method. The DUS characteristic that distinguished most pairs of varieties was ‘Plant: time of inflorescence emergence’, especially when QTL were included in the model (39%). Other characteristics with potentially useful levels of discrimination included ‘Plant: vegetative growth habit (after vernalization)’, ‘Plant: height (after vernalization)’, ‘Plant: natural height at inflorescence emergence’, ‘Plant: growth habit at inflorescence emergence’ (particularly for tetraploids), ‘Flag leaf: width’, ‘Plant: length of longest stem, inflorescence included (when fully expanded)’, and ‘Inflorescence: length’ (for tetraploids).

The performance of gBLUP was clearly related to the proportion of differences found by COYD, which in turn will be related to heritability for the characteristic. As expected, fewer differences are found by gBLUP, reflecting the cross-validated nature of the prediction. Many DUS characteristics had low proportions of distinguished varieties even with COYD. The benefit of adding in specific markers identified by GWAS in a gBLUP + QTL model was most pronounced for ‘Plant: time of inflorescence emergence’, evidenced by an increased number of variety pairs found to be Distinct (Table 1), although R^2^ values were not greatly improved (*R*^2^ values of 93.17%).

Within current DUS testing approaches, Distinctness decisions are made over all DUS characteristics, with Distinctness required in only one characteristic. For perennial ryegrass, the proportions of pairs of simulated ‘candidate’ and ‘known’ varieties that were identified as Distinct based on at least one DUS characteristic are summarised (Table 2). These are compared with the number distinguished using the long-term version of COYD (based on a three-year criterion). On average, approximately 40% of known varieties were distinguished from each ‘candidate’ using gBLUP, and 50% for gBLUP + QTL. Note that COYD does not achieve discrimination between 100% of pairs of varieties (95% for diploids and 89% for tetraploids). This may be explained in part by the use of a long-term version of COYD on historical data, whereas distinctness decisions would have been made based on three-year series of trials.

**Table 2:** Perennial ryegrass: proportion of pairs of simulated ‘candidates’ and ‘known’ varieties distinguished by cross-validated genomic prediction over all quantitative DUS characteristics. In the cross-validation scheme, ‘candidates’ only have genetic data whereas ‘known’ varieties have both phenotypic and genetic data

Method	Diploid (%)	Tetraploid (%)
gBLUP	41	41
gBLUP + QTL	52	51
COYD	95	89

Results for the gBLUP and gBLUP + QTL methods are compared to long-term COYD

Whilst the DUS trial size reduction for perennial ryegrass estimated by simulation was considerable for one or two ‘candidates’, for more ‘candidates’ the benefit becomes less pronounced (Table 3), and compares poorly with alternatives such as cyclic planting (UPOV 2022), which can eliminate approximately 1/3 of known varieties from the growing trial.

**Table 3:** Perennial ryegrass: estimates of DUS trial size reduction from the use of genomic prediction, based on simulation

Number of candidates	Diploid (%)	Tetraploid (%)
1	52	52
2	31	31
5	12	13
10	6	7

This represents the proportion of the known varieties that would not need to be planted

#### Wheat

Residual plots for each characteristic based on the gBLUP model without QTLs are given in the Supplementary Information (histogram of residual, normal Q-Q plot, residuals vs fitted value). All characteristics showed clear signs of shrinkage, due to the lack of replication. Despite the ordinal scale of the responses, many of the characteristics had reasonable residuals. Others though showed deviations from assumptions. These include ‘Plant: frequency of plants with recurved flag leaves’ and ‘Shoulder shape’ (fat tails), and ‘Glaucosity of flag leaf sheath’ (left skewed).

**Fig. 3:**
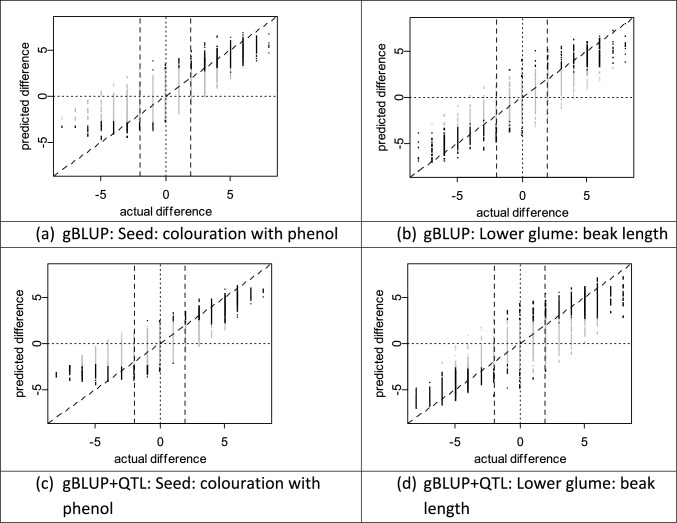
Wheat: simulated use of genomic prediction to identify distinct pairs of varieties for two selected DUS characteristics. The vertical axis represents the differences in predicted mean expression between simulated ‘candidates’, whose field data is ignored, and all other varieties in the historical DUS data set. The horizontal axis presents the differences between the same pairs of varieties, but based on the phenotypic data. Points on the plots represent differences in means between a pair of varieties. The black points are those where genomic prediction finds the candidate and known variety to be distinct (with probability 0.01) and grey points represent those that were not found Distinct. The vertical dashed lines represent a two-point difference on the 1 to 9 scale. The left-hand panels are for DUS characteristic ‘seed colouration with phenol’, and the right-hand panels are for ‘beak length’. The upper panels used gBLUP, whereas the lower panels used gBLUP + QTL

The results of the cross-validation scheme for simulating real-life application of genomic prediction to the wheat historical DUS data are illustrated for two wheat DUS characteristics (Fig. 3). Both characteristics were predicted with reasonable accuracy (R^2^ values of 62.59% and 74.89% for ‘Seed: colouration with phenol’ and ‘Lower glume: beak length’ respectively), with gBLUP + QTL providing superior performance (corresponding R^2^ values of 74.15% and 80.27%).

The wheat results for the 19 quantitative DUS characteristics analysed are summarised in Table 4, which shows the proportion of pairs of varieties (simulated candidates and known varieties) distinguished by genomic prediction, as well as by the actual difference in characteristic score. For all characteristics, results are shown for the gBLUP method, and for those six characteristics with SNPs potentially linked to QTLs as identified by Zanella et al. (2026), results are given for the gBLUP + QTL method. Good levels of discrimination with genomic prediction were found for four DUS characteristics: ‘Seed: colouration with phenol’, ‘Plant: frequency of plants with recurved flag leaves’, ‘Ear: length of scurs or awns’, and ‘Lower glume: length of beak’. The addition of QTLs improved discrimination throughout, and markedly so for ‘Seed: colouration with phenol’, ‘Ear: length of scurs or awns’, and ‘Lower glume: length of beak’.

**Table 4:** Wheat: proportion of pairs of simulated ‘candidates’ and ‘known’ varieties distinguished by cross-validated genomic prediction, compared with actual differences based on DUS data alone (with a threshold of two notes)

Characteristic	Actual difference (based on DUS data) (%)	gBLUP (%)	gBLUP + QTL (%)
Seed: colouration with phenol	45.0	5.8	16.3
Coleoptile: anthocyanin colouration	56.7	1.7	5.9
Plant: growth habit	35.7	1.4	
Plant: frequency of plants with recurved flag leaves	40.2	7.1	
Ear: Time of emergence	42.4	2.6	
Flag leaf: glaucosity of sheath	29.7	1.0	
Flag leaf: glaucosity of blade	47.7	3.8	
Ear: glaucosity	42.1	1.6	
Culm: glaucosity of neck	36.3	1.1	
Plant: length	44.9	4.2	
Ear: density	34.7	2.3	2.9
Ear: length	36.9	3.7	
Ear: length of scurs or awns	61.6	11.9	23.3
Apical rachis segment: area of hairiness on convex surface	57.6	1.1	
Lower glume: shoulder width	42.1	1.3	
Lower glume: shoulder shape	42.6	0.1	
Lower glume: length of beak	58.1	13.8	19.8
Lower glume: shape of beak	47.9	0.9	
Lower glume: area of hairiness on internal surface	57.6	2.5	4.5

In the cross-validation scheme, ‘candidates’ only have genetic data whereas ‘known’ varieties have both phenotypic and genetic data. Note for wheat, there was only one value supplied for each variety

Cumulating over these quantitative characteristics, the proportions of varieties discriminated was 38% for gBLUP and 55% for gBLUP + QTL, which was similar to perennial ryegrass. It is noteworthy that the apparent discrimination levels using actual DUS phenotypic data were high for all DUS characteristics. However, the association between the proportions of gBLUP differences and actual differences was not clear. Whilst two of the most discriminating characteristics based on actual differences, ‘Ear: length of scurs or awns’, and ‘Ear: length of scurs or awns’ gave good discrimination also with gBLUP, other highly discriminating characteristics, such as ‘Apical rachis segment: area of hairiness on convex surface’, did not work as well with gBLUP.

### Performance of COYD-GP for distinctness assessment in perennial ryegrass

The performance of the proposed method extending the COYD to include information from markers, COYD-GP, was evaluated based on the perennial ryegrass data set. The proportion of differences between varieties found by long-term versions of COYD and COYD-GP were compared (Table 5). COYD-GP produced increases in the proportion of differences found between varieties in all DUS characteristics, with increases of up to 10% (for ‘growth habit at inflorescence emergence’ with tetraploid varieties).

**Table 5:** Percentage of varieties found distinct by long-term COYD and COYD-GP for perennial ryegrass

Ryegrass DUS Characteristic	Diploid		Tetraploid	
	COYD (%)	COYD-GP (%)	COYD (%)	COYD-GP (%)
Plant: vegetative growth habit (without vernalization)	28.8	30.6	10.1	11.5
Leaf: intensity of green colour (without vernalization)	7.7	9.9	2.7	3.2
Plant: width (after vernalization)	25.3	27.3	15.3	17.0
Plant: vegetative growth habit (after vernalization)	37.0	39.6	21.2	27.8
Plant: height (after vernalization)	46.8	48.8	24.2	30.1
Leaf: intensity of green colour (after vernalization)	17.0	18.5	11.9	16.8
Plant: time of inflorescence emergence	77.5	77.8	70.0	70.9
Plant: natural height at inflorescence emergence	39.0	41.2	25.3	29.9
Plant: growth habit at inflorescence emergence	15.7	19.8	22.0	31.8
Flag leaf: length	13.5	15.7	9.8	13.3
Flag leaf: width	39.8	41.9	28.0	33.0
Flag leaf: length/ width ratio	26.7	29.6	9.1	12.4
Plant: length of longest stem, inflorescence included (when fully expanded)	43.3	46.2	31.5	37.0
Plant: length of upper internode	17.6	20.9	6.4	8.3
Inflorescence: length	28.0	28.7	27.9	31.3
Inflorescence: number of spikelets	37.5	38.8	20.0	22.4
Inflorescence: density	29.7	31.0	29.4	32.6
Inflorescence: length of outer glume on basal spikelet	26.2	27.8	16.4	20.0
Inflorescence: length of basal spikelet excluding awn	22.2	25.1	16.8	20.9
Inflorescence: spikelet protuberance	22.4	25.8	15.7	21.1
Inflorescence: glume span	30.3	33.3	14.4	17.3

COYD is based only on phenotypic data, where COYD-GP also includes genetic information

## Discussion

This paper proposes two novel uses of genomic prediction in the context of DUS assessment of new plant varieties: for the planning of DUS growing trials; and for direct assessment of Distinctness once the growing trials have been carried out.

Genetic markers have long been considered as a way to manage DUS trials, providing a mechanism for selecting existing varieties to compare with the candidate varieties. The proposed framework for Reference Collection management provides a more targeted approach for quantitative characteristics than existing examples of UPOV application model b). It is anticipated to surpass the current methods for the following reasons:

- It is expected to yield more precise predictions for individual DUS characteristics based on genomic predictions, in comparison to the typical correlations observed between phenotypic and genetic distances.
- It takes advantage of the principle that Distinctness between a candidate and a known variety is required in only one DUS characteristic.

The framework gives greater potential for efficient DUS trial management, especially if combined with UPOV application model a) for qualitative characteristics.

Although the use of genetic markers has been accepted for planning of trials, the only current accepted method for applying them for direct Distinctness assessment requires an exact prediction of phenotype. This is only applicable in a relatively small number of cases. We claim that our new approach, COYD-GP, using genomic prediction, is consistent with the UPOV principles for DUS, and will provide more precise determinations of Distinctness for measured quantitative DUS characteristics in cross-pollinated crops, where the current method COYD is already used. However, the validity of this approach will need to be discussed by DUS experts in UPOV fora. Like COYD, Distinctness using COYD-GP is based on comparisons of varieties for individual DUS characteristics. The addition of genetic relationship information simply makes the estimation of phenotypic effects more precise, given uncertainty caused by genotype-by-environment interaction. This provides a COYD-style approach with better information with which to make decisions in cross-pollinated crops. The introduction of genetic markers for improving the precision of varietal differences helps to confer COYD-GP a significant advantage over COYD, as demonstrated with the ryegrass DUS data. If markers are used for other purposes, such as Reference Collection management, then their use here would come at no extra cost. The COYD-GP could be used to provide supporting evidence when otherwise there is doubt on the case for distinctness.

In the evaluation of the new approach for Reference Collection management for perennial ryegrass where the focus is on trial size reduction by identification of reference varieties that are clearly different from the candidates, our simulations indicated that where DUS characteristics have relatively low levels of heritability, the method might not be able to discriminate a sufficient number of variety pairs to provide a distinct advantage over alternatives such as cyclic planting (which would result in a trial size reduction of one third through a rotation of known varieties across three years of trials). However, these simulation results are likely to be pessimistic as actual candidate varieties exhibit greater similarity to each other than our simulated random selection (and which have all been considered Distinct in a DUS test). A potentially useful output of the genomic prediction analysis was the demonstration that timing of inflorescence emergence can be predicted accurately through genetic markers. This relationship could be useful in the management of both DUS and performance trials, as well as for the adjustment of yield and forage nutritive value for maturity in the latter trials.

In the case of wheat where the focus is on identifying similar reference varieties to candidates, the results demonstrated potential for the planning of DUS trials. Good predictions of phenotypic similarity were exhibited by three quantitative DUS characteristics. The overall efficacy of the approach should be substantially improved if based on more comprehensive data (i.e., raw measurements where available, and yearly scores were not consolidated over years), and with the inclusion of qualitative characteristics under UPOV application model a). In the case of the latter, the wheat GWAS results found strong marker relationships for results for ‘Ear: scurs or awns’, and for ‘Seasonal type’ (Zanella et al. 2026). Extension of the gBLUP method to more effectively address ordinal data would likely increase the approach’s effectiveness.

The usefulness of the genomic prediction approach (and for UPOV application model b)) for Reference Collection management is clearly contingent upon the strength of the relationship between the phenotypic characteristics and the genetic markers. This relationship is, at least partially, dependent on the heritability of the DUS characteristics. A greater number of DUS characteristics with high heritability will increase the number of known varieties that the candidate can be considered Distinct from. The study was limited by the relatively low number of varieties available. An increased number of varieties, representing a broader genetic pool, would likely yield improved results. It is well established that the efficacy of genomic prediction is contingent on sufficient data. In the case of perennial ryegrass, another caveat is that the genomic prediction models were developed utilising varietal allele frequencies in addition to varietal phenotype means. It may be expected that superior prediction models and marker-trait associations could be established with genotypes and phenotypes from individual plants. Nevertheless, family and varietal allele frequencies have been utilised frequently for advancing genomic prediction in perennial ryegrass breeding (Pembleton et al. 2018; Fè et al. 2016; Konkolewska et al. 2024), and the cost effectiveness of genotyping varietal pools is also likely to be attractive in the case of DUS testing.

We anticipate that genomic prediction for trial management can be used either to reduce DUS trial size, mainly for agricultural cross-pollinated crops such as perennial ryegrass, or to identify similar varieties to enable proximal planting for more precise field comparisons, as in the second example given under UPOV application model b) (UPOV 2020a). For instance, in wheat in the UK, second year candidates are planted alongside similar known varieties based on DUS information from the first year. The identification of these similar varieties cannot happen for the first year of test due to the absence of prior information, and a third DUS trial year may be required if the distinctness assessment is inconclusive after the second year. The genomic prediction approach should facilitate more effective selection of similar known varieties for planting alongside candidates in the first year and therefore reduce or remove the requirement for a third year of testing.

Candidates approaching their second year of DUS testing possess both field data (for one year) and genetic marker data. In such instances, predictions of Distinctness or similarity will be predicated on a more comprehensive information set, thereby potentially facilitating the identification of a greater number of Distinct variety pairs at this stage or, alternatively, a more refined selection of similar varieties depending on the trial management approach for the crop. This approach could be utilised for the strategic planning of second-year components of DUS trials.

The primary focus of this work has been on the framework rather than specific genetic marker technologies or genomic prediction methodology. There exists potential for improvement on the latter in the context of DUS data. The optimum method will depend on several factors (Lourenço et al. 2024), including the genetic architecture of the particular trait. The size of the data set may restrict the complexity of the model. Some methods are more computationally demanding, which may constrain evaluation by cross-validation. It is important that the method be capable of incorporating data structure (e.g. trial effects) and provide measures of precision alongside predictions. Those methods that can be expressed as hierarchical mixed models possess an advantage in this regard. Bayesian approaches offer one way forward, but require care with convergence. Machine learning approaches offer another route; however, it may be difficult to adequately account for data structure and to estimate standard errors. Both Bayesian and machine learning add computational time costs, but these may be ameliorated with more powerful computers with parallel processing facilities.

The method employed here was based on linear mixed models. We note that mixed model predictions for contrasts of random effects do not account for uncertainty in the estimation of variance components (Forkman and Piepho 2013). Predictions should be adequate if the variance components are well estimated. This depends on having sufficiently sized and structured historical data sets. Alternatively, a Bayesian form of the same model would avoid such concerns. Whichever genomic prediction method is used, it is important to assess its performance fully using historical data. This would include consideration of type I and II errors associated with decisions. This work did not assess these errors, as failed candidates were excluded from the data sets due to data access restrictions.

Whilst this study has employed a method suitable for a normal response, DUS characteristics often utilise other scales, such ordinal, nominal, and binary. The ordinal scale is particularly common in DUS, with the standard 1 to 9 scale in UPOV guidelines, and measurements are frequently made directly on this scale. As Laidig et al (2021) note, in variety trials, it is common practice to analyse ordinal response data as continuous, and given no gross departures from assumptions this may be reasonable. However, in the DUS context it would be particularly advantageous to make predictions on the ordinal scale, as this more closely relates to how decisions are made. Gambarota and Altoè (2024) point out the benefits of modelling ordinal data as ordinal in a general context, and Kizilkaya et al (2014) demonstrate greater prediction accuracy for ordinal genomic prediction especially when training sets are small. There are a number of different models for ordinal responses (Agresti 2010; Tutz 2021), with the mostly applied being the cumulative model with either logit (proportional odds) or probit link functions. For frequentist mixed model-based approaches, such as gBLUP and gBLUP + QTL, there was not thought to be a theoretical obstacle to an ordinal-based approach, but to date, no functionality in R has been found. It may be possible to extend one of the existing R packages for a frequentist ordinal linear mixed model, such as ‘ordinal’, to deal with genomic data. Instead, we suggest that it would be better to take a Bayesian approach where there are existing methods for modelling ordinal responses with genomic data (Montesinos-López et al. 2015), despite the added computational costs. Indeed, given that, as in the case of the wheat example here, the level of replication is low, Bayesian methods may have advantages (Forkman and Piepho 2013) over empirical BLUP. The R package ‘brms’ (Bürkner 2017) offers a wide range of ordinal models, and can model genetic relationships. For ordinal characteristics, rather than comparing to COYD thresholds, it is more appropriate to use genomic prediction to assess the probability of exceeding the threshold for Distinctness in a particular DUS characteristic (which is commonly two points on the 1 to 9 scale). For binary- or nominal-scaled DUS characteristics, UPOV application model a) is more appropriate. Some characteristics are classified as pseudo-qualitative (UPOV 2002). This classification applies when the scale is at least partly continuous, but varies in more than one dimension. Examples include descriptions of two-dimensional shapes. Genomic prediction could be applied to these types of characteristics with caution, potentially by separating the dimensions that differentiate the shapes described.

In conclusion, we have proposed and demonstrated the use of genomic prediction for DUS trial management. This should outperform the current examples accepted in UPOV application model (b), and could be used in tandem with examples accepted in UPOV application model (a) (UPOV 2020a). The way that predictions from this approach are used in practice for trial management will depend on the crop. Following studies should look at optimising the way that DUS trials are designed using genomic prediction, including the use of these predictions to identify close pairs of varieties that can be compared from the first year of trials. Different genomic prediction methods should be compared, with a view to identify good choices for different crops and characteristics. Such studies should have access to all varieties tested, including candidates, in order to give a fuller basis for assessment of effectiveness. We have also proposed a new genomic prediction-based method for assessing distinctness in cross-pollinated crops. This provides advantages in effectiveness over the existing COYD method, but its validity and utility need to be considered by DUS experts in UPOV fora.

## Supplementary Information

Below is the link to the electronic supplementary material.

Supplementary file1 (PDF 2465 KB)

Supplementary file2 (PDF 979 KB)

Supplementary file3 (XLSX 342 KB)

Supplementary file4 (DOCX 25 KB)

## Data Availability

The genome resequencing reads for all perennial ryegrass cultivars utilised in this study have been deposited into the NCBI sequence read archive (SRA) under the BioProject ID: PRJNA1255563. Phenotypic data for perennial ryegrass is supplied in the Supplementary Information. The data for the wheat example is referenced in Zanella et al. (2026). All data is anonymised.
