# Supplementary material for: Integrating genomic prediction into crop DUS testing: new approaches in support of reference collection management and distinctness assessment
Source: Theor Appl Genet. 2026 Mar 12;139(4):93. doi: 10.1007/s00122-026-05198-6 (PMC12982246; doi:10.1007/s00122-026-05198-6)
Supplement: Supplementary file 1 — Supplementary file1 (PDF 2465 KB) [file 122_2026_5198_MOESM1_ESM.pdf]

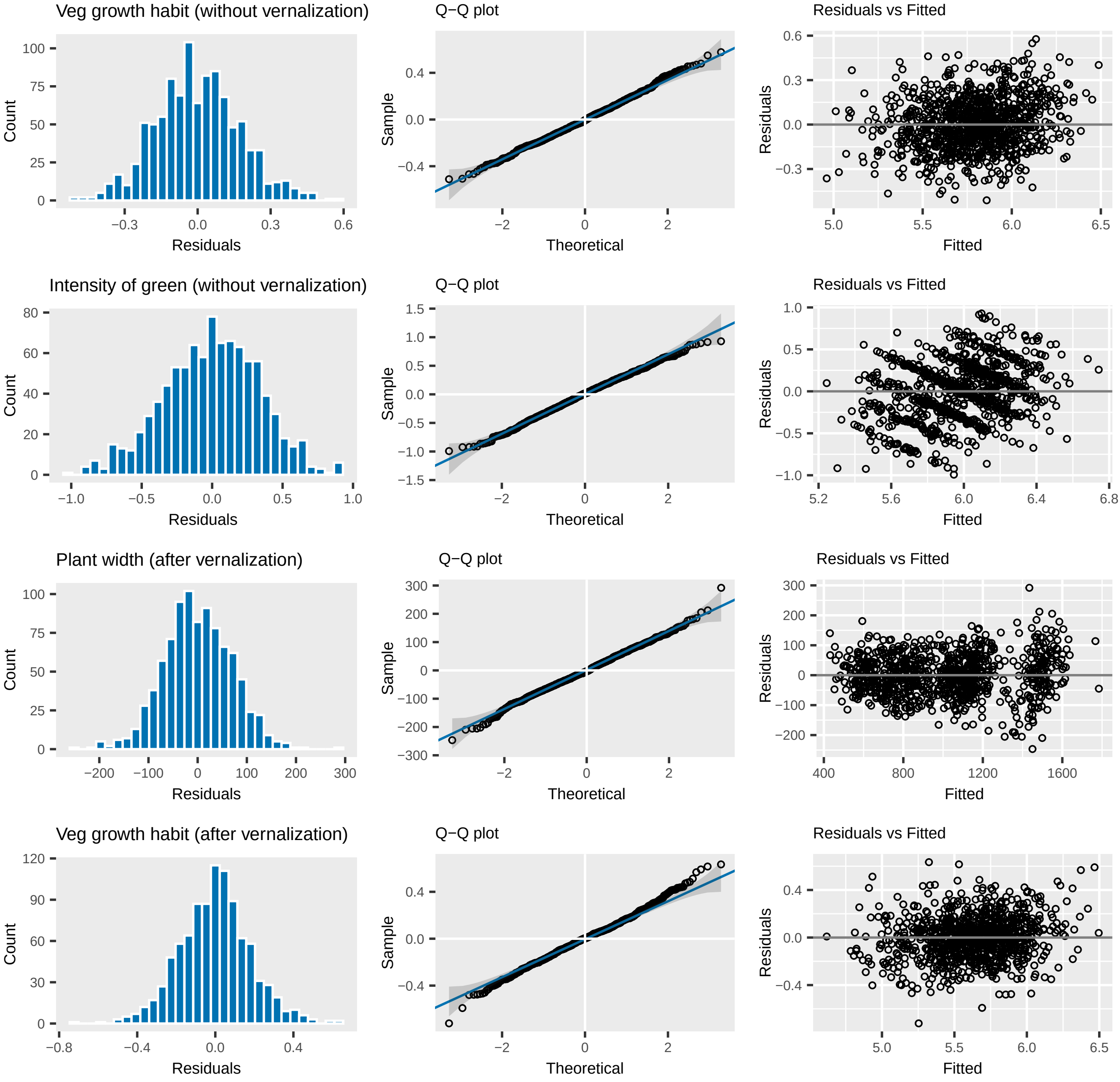
Veg growth habit (without vernalization)
Count
Residuals
Q−Q plot
Sample
Theoretical
Residuals vs Fitted
Residuals
Fitted
Intensity of green (without vernalization)
Count
Residuals
Q−Q plot
Sample
Theoretical
Residuals vs Fitted
Residuals
Fitted
Plant width (after vernalization)
Count
Residuals
Q−Q plot
Sample
Theoretical
Residuals vs Fitted
Residuals
Fitted
Veg growth habit (after vernalization)
Count
Residuals
Q−Q plot
Sample
Theoretical
Residuals vs Fitted
Residuals
Fitted

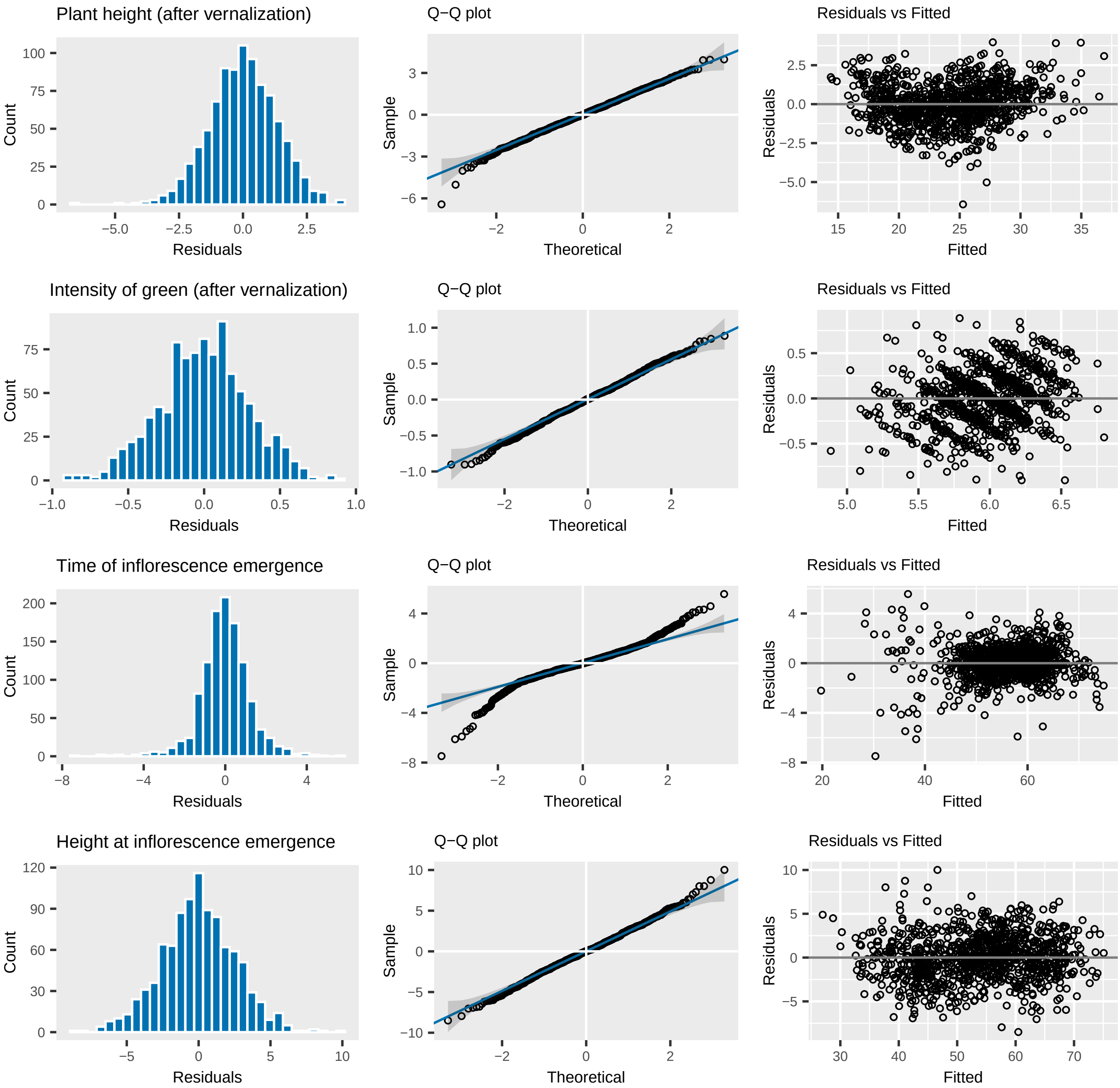
Plant height (after vernalization)
Q−Q plot
Residuals vs Fitted
Intensity of green (after vernalization)
Q−Q plot
Residuals vs Fitted
Time of inflorescence emergence
Q−Q plot
Residuals vs Fitted
Height at inflorescence emergence
Q−Q plot
Residuals vs Fitted
Count
Residuals
Sample
Theoretical
Residuals
Fitted

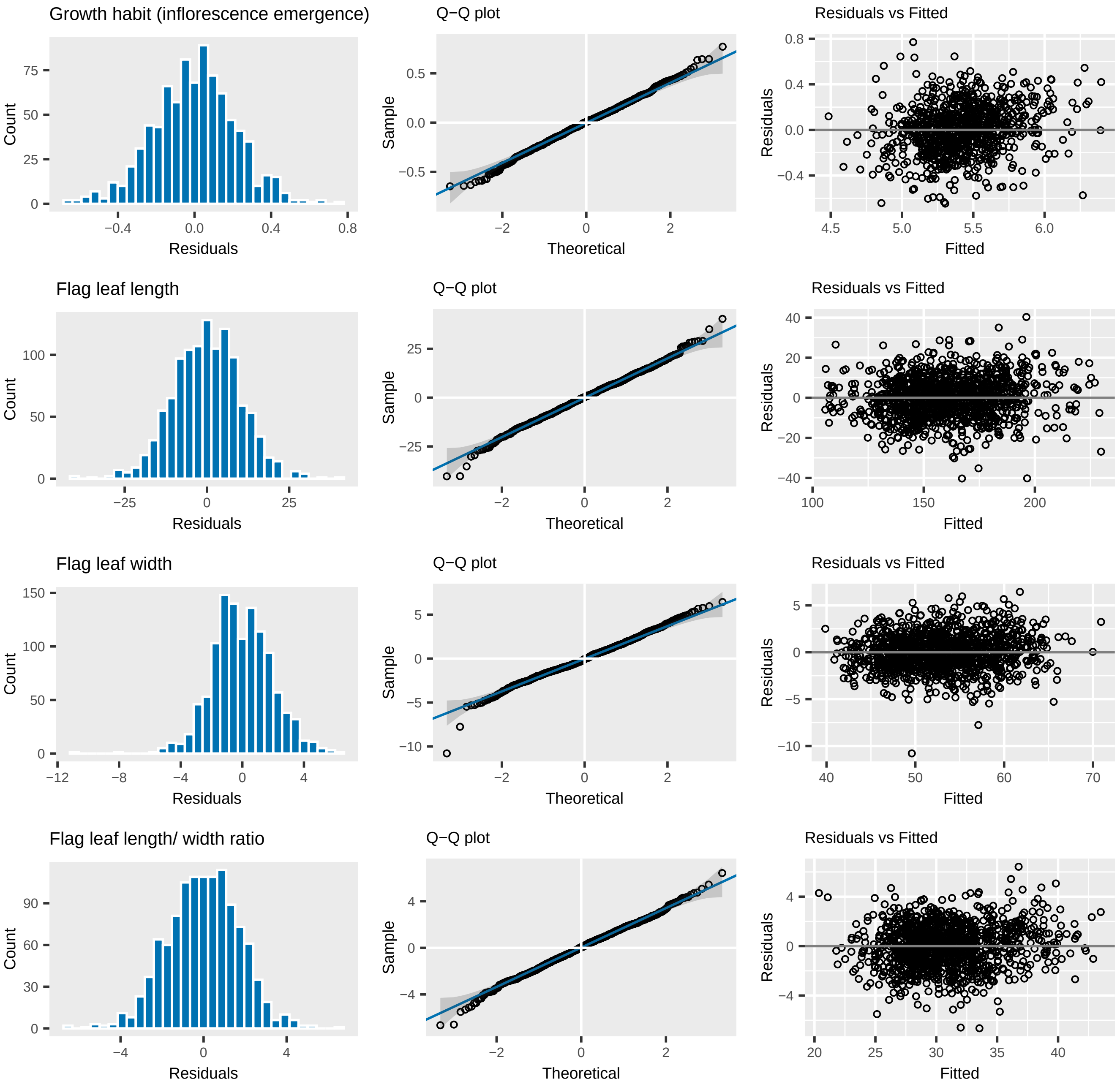
Growth habit (inflorescence emergence)
Count
Residuals
Q−Q plot
Sample
Theoretical
Residuals vs Fitted
Residuals
Fitted
Flag leaf length
Count
Residuals
Q−Q plot
Sample
Theoretical
Residuals vs Fitted
Residuals
Fitted
Flag leaf width
Count
Residuals
Q−Q plot
Sample
Theoretical
Residuals vs Fitted
Residuals
Fitted
Flag leaf length/ width ratio
Count
Residuals
Q−Q plot
Sample
Theoretical
Residuals vs Fitted
Residuals
Fitted

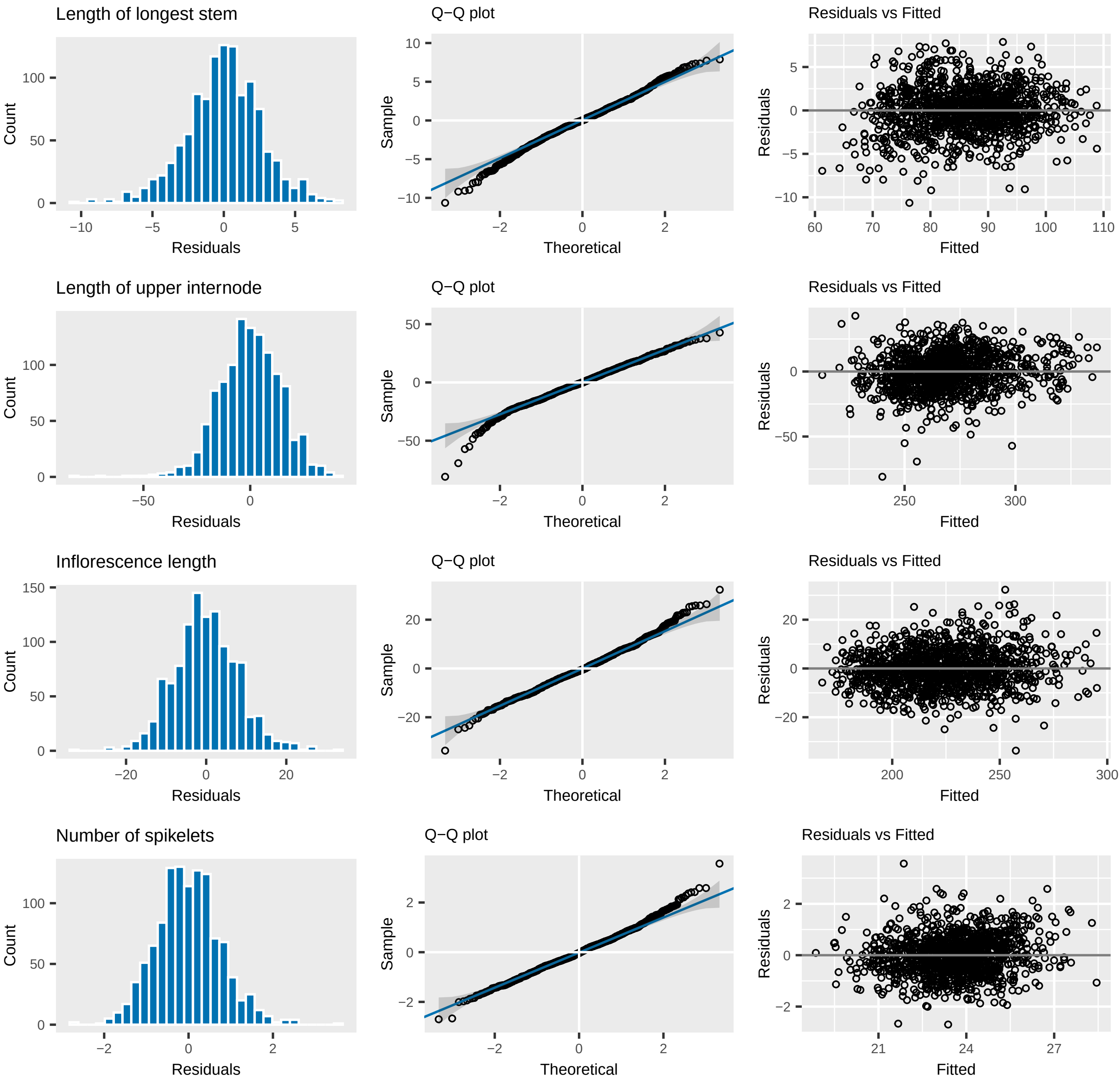
Length of longest stem
Count
Residuals
Q−Q plot
Sample
Theoretical
Residuals vs Fitted
Residuals
Fitted
Length of upper internode
Count
Residuals
Q−Q plot
Sample
Theoretical
Residuals vs Fitted
Residuals
Fitted
Inflorescence length
Count
Residuals
Q−Q plot
Sample
Theoretical
Residuals vs Fitted
Residuals
Fitted
Number of spikelets
Count
Residuals
Q−Q plot
Sample
Theoretical
Residuals vs Fitted
Residuals
Fitted

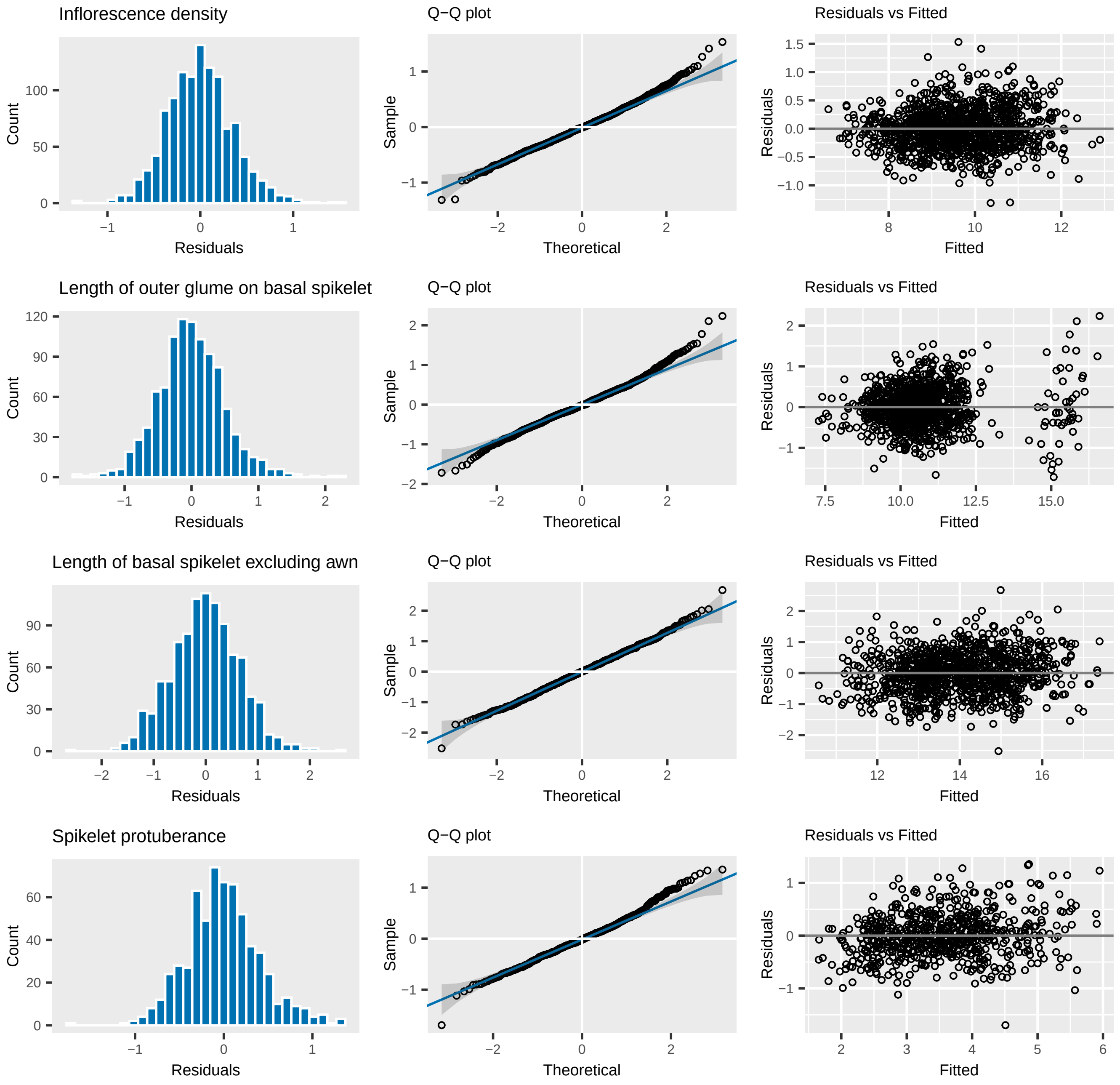
Inflorescence density
Q−Q plot
Residuals vs Fitted
Length of outer glume on basal spikelet
Q−Q plot
Residuals vs Fitted
Length of basal spikelet excluding awn
Q−Q plot
Residuals vs Fitted
Spikelet protuberance
Q−Q plot
Residuals vs Fitted
Count
Residuals
Sample
Theoretical
Residuals
Fitted

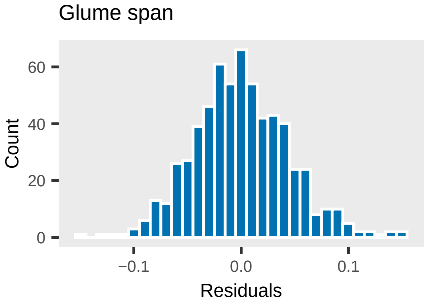
Glume span
Count
60
40
20
0
−0.1
0.0
0.1
Residuals

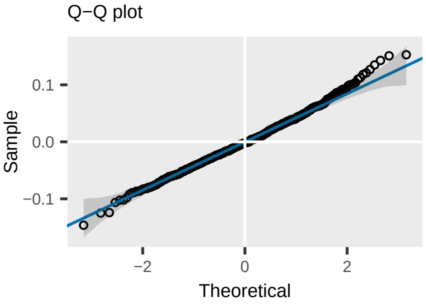
Q−Q plot
Sample
0.1
0.0
−0.1
−2
0
2
Theoretical

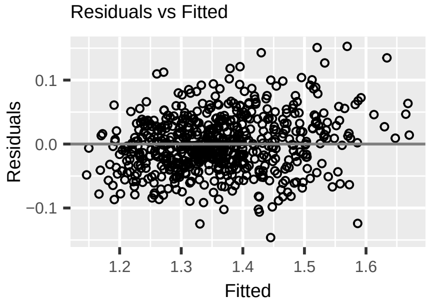
Residuals vs Fitted
Residuals
0.1
0.0
−0.1
1.2
1.3
1.4
1.5
1.6
Fitted
