# Supplementary material for: Integrating genomic prediction into crop DUS testing: new approaches in support of reference collection management and distinctness assessment
Source: Theor Appl Genet. 2026 Mar 12;139(4):93. doi: 10.1007/s00122-026-05198-6 (PMC12982246; doi:10.1007/s00122-026-05198-6)
Supplement: Supplementary file 2 — Supplementary file2 (PDF 979 KB) [file 122_2026_5198_MOESM2_ESM.pdf]

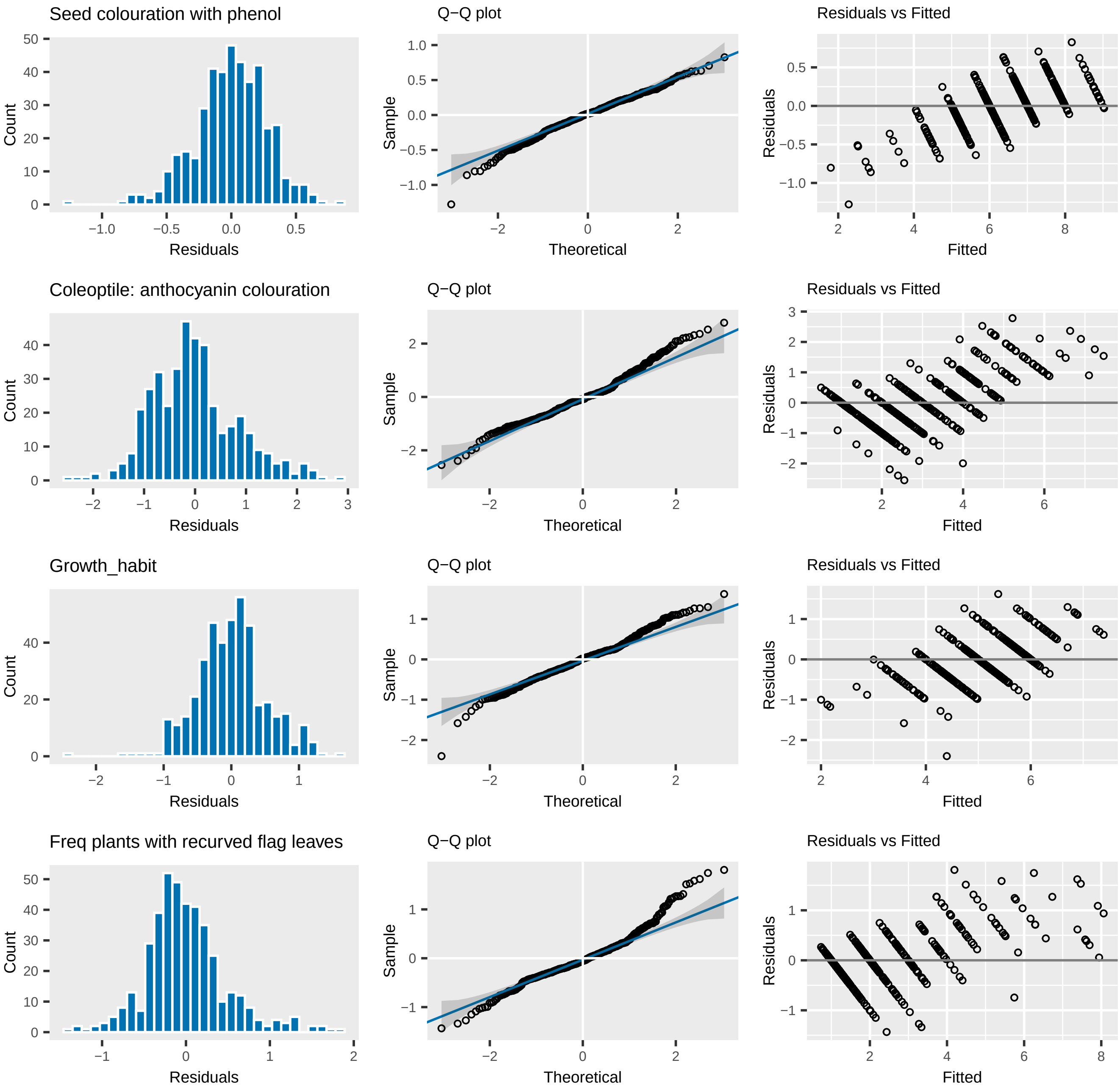
Seed colouration with phenol
Count
Residuals
Q−Q plot
Sample
Theoretical
Residuals vs Fitted
Residuals
Fitted
Coleoptile: anthocyanin colouration
Count
Residuals
Q−Q plot
Sample
Theoretical
Residuals vs Fitted
Residuals
Fitted
Growth_habit
Count
Residuals
Q−Q plot
Sample
Theoretical
Residuals vs Fitted
Residuals
Fitted
Freq plants with recurved flag leaves
Count
Residuals
Q−Q plot
Sample
Theoretical
Residuals vs Fitted
Residuals
Fitted

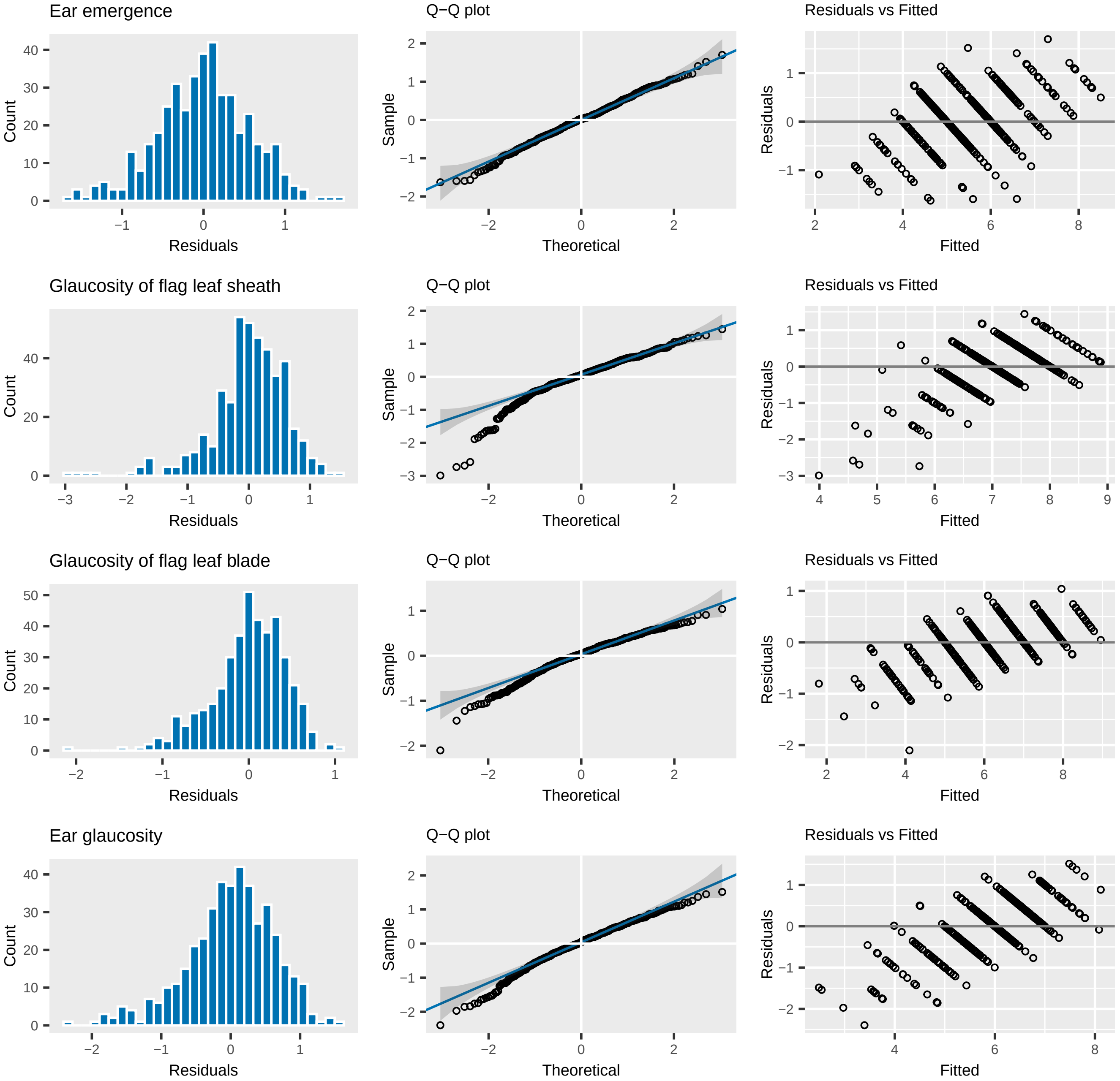
Ear emergence
Count
Residuals
Q−Q plot
Sample
Theoretical
Residuals vs Fitted
Residuals
Fitted
Glaucosity of flag leaf sheath
Count
Residuals
Q−Q plot
Sample
Theoretical
Residuals vs Fitted
Residuals
Fitted
Glaucosity of flag leaf blade
Count
Residuals
Q−Q plot
Sample
Theoretical
Residuals vs Fitted
Residuals
Fitted
Ear glaucosity
Count
Residuals
Q−Q plot
Sample
Theoretical
Residuals vs Fitted
Residuals
Fitted

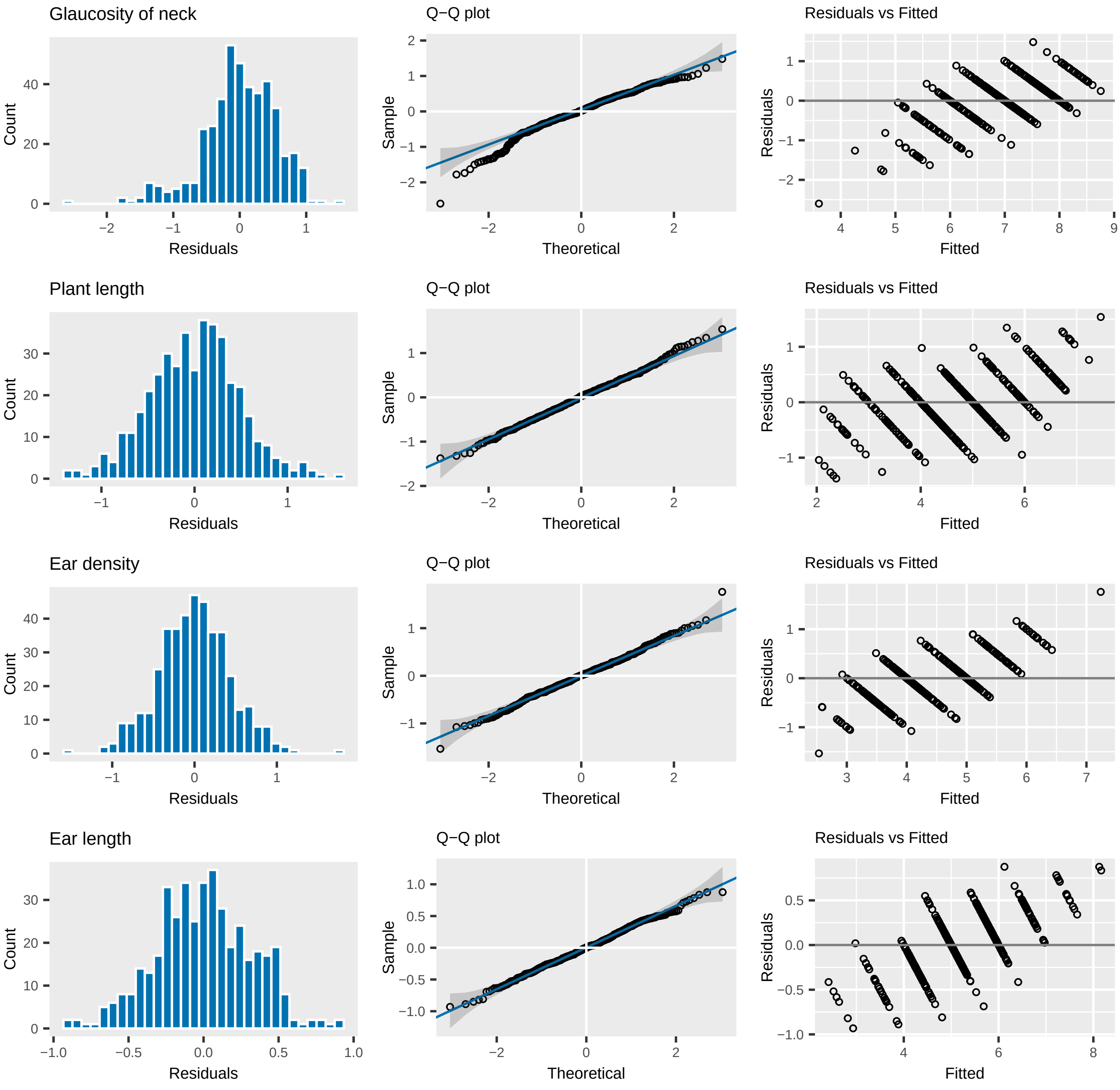
Glaucosity of neck
Count
Residuals
Q−Q plot
Sample
Theoretical
Residuals vs Fitted
Residuals
Fitted
Plant length
Count
Residuals
Q−Q plot
Sample
Theoretical
Residuals vs Fitted
Residuals
Fitted
Ear density
Count
Residuals
Q−Q plot
Sample
Theoretical
Residuals vs Fitted
Residuals
Fitted
Ear length
Count
Residuals
Q−Q plot
Sample
Theoretical
Residuals vs Fitted
Residuals
Fitted

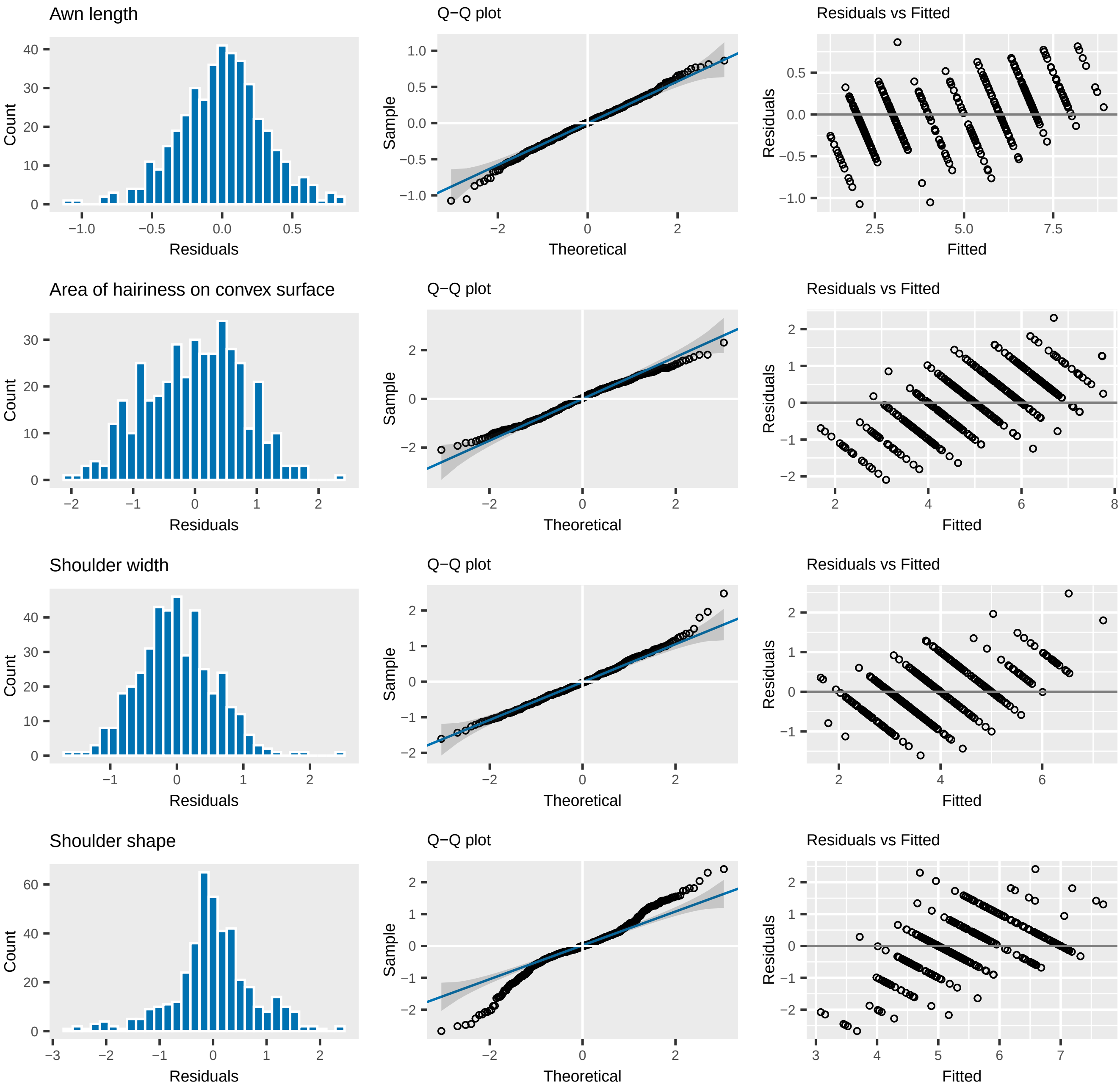

Awn length
Count
Residuals
Q−Q plot
Sample
Theoretical
Residuals vs Fitted
Residuals
Fitted
Area of hairiness on convex surface
Count
Residuals
Q−Q plot
Sample
Theoretical
Residuals vs Fitted
Residuals
Fitted
Shoulder width
Count
Residuals
Q−Q plot
Sample
Theoretical
Residuals vs Fitted
Residuals
Fitted
Shoulder shape
Count
Residuals
Q−Q plot
Sample
Theoretical
Residuals vs Fitted
Residuals
Fitted

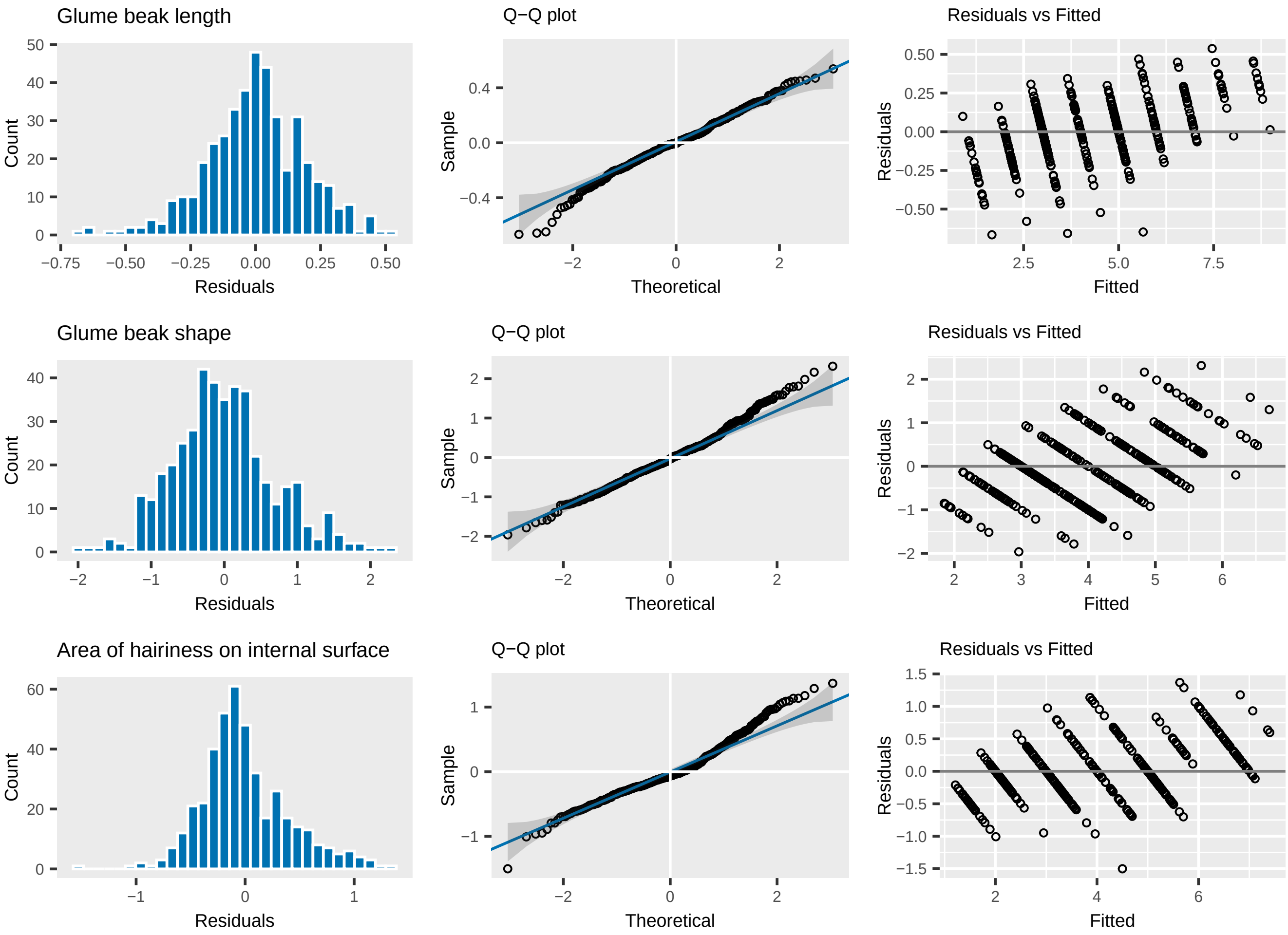
Glume beak length
Q−Q plot
Residuals vs Fitted
Count
Residuals
Sample
Theoretical
Fitted
Glume beak shape
Q−Q plot
Residuals vs Fitted
Count
Residuals
Sample
Theoretical
Fitted
Area of hairiness on internal surface
Q−Q plot
Residuals vs Fitted
Count
Residuals
Sample
Theoretical
Fitted
