# Supplementary material for: Integrating genomic prediction into crop DUS testing: new approaches in support of reference collection management and distinctness assessment
Source: Theor Appl Genet. 2026 Mar 12;139(4):93. doi: 10.1007/s00122-026-05198-6 (PMC12982246; doi:10.1007/s00122-026-05198-6)
Supplement: Supplementary file 4 — Supplementary file4 (DOCX 25 KB) [file 122_2026_5198_MOESM4_ESM.docx]

# Supplementary Information

**Table S1 Perennial ryegrass SNPs with significant GWAS associations with quantitatively measured DUS traits**

| DUS characteristic | Most significant SNP^[[1]](#footnote-1)^ | Chromosome |  | -log_10_(p-value) |
| --- | --- | --- | --- | --- |
| Plant: width (after vernalization) | *Lp_chr1_0_82655397* | 1 |  | 6.4 |
| Plant: vegetative growth habit (after vernalization) | *Lp_chr7_0.1_160500269* | 7 |  | 6.1 |
| Plant: height (after vernalization) | *Lp_chr7_0.1_160500269* | 7 |  | 6.2 |
| Plant: time of inflorescence emergence | *Lp_chr1_0_172971804* | 1 |  | 6.8 |
|  | *Lp_chr2_0_8841608* | 2 |  | 7.6 |
|  | *Lp_chr7_0.1_29708865* | 7 |  | 20.0 |
| Flag leaf: length/ width ratio | *Lp_chr1_0_37108431* | 1 |  | 6.5 |

**Table S2 Wheat SNPs with significant GWAS associations with quantitatively measured DUS traits (Zanella et al. XXX)**

| Wheat DUS characteristic | Most significant SNP | Chromosome | Position (Mbp) | -log_10_(p-value) |
| --- | --- | --- | --- | --- |
| Seed: colouration with phenol | *AX.643826080* | 2A | 712.7214 | 26.7 |
|  | *AX.94687651* | 2A | 712.5551 | 44.8 |
| Coleoptile: anthocyanin colouration | *AX.95229802* | 7A | 118.3269 | 16.6 |
|  | *AX.94969837* | 7A | 126.8674 | 15.7 |
| Ear density | *AX.94758742* | 5B | 514.8295 | 6.5 |
|  | *AX.94382460* | 5B | 514.2887 | 6.5 |
| Awn or scur length | *AX.94613491* | 5A | 698.5097 | 26.3110.9 |
|  | *AX.110052584* | 5A | 698.5651 | 21.0 |
| Lower glume: beak length | *AX.94613491* | 5A | 698.5097 | 26.1 |
|  | *AX.643813032* | 5A | 694.5193 | 10.4 |
| Lower glume: area of hairiness on internal surface | *AX.643804373* | 4D | 502.7278 | 8.4 |

1. Position in the genome is encoded in the SNP name. So, in the example Lp_chr1_0_82655397, it would be 82.655 Mbp on chromosome 1 [↑](#footnote-ref-1)
